# Long-chain noncoding RNA-GAS5/hsa-miR-138-5p attenuates high glucose-induced cardiomyocyte damage by targeting CYP11B2

**DOI:** 10.1042/BSR20202232

**Published:** 2021-09-03

**Authors:** Xiaozhen Zhuo, Kai Bai, Yingxian Wang, Peining Liu, Wen Xi, Jianqing She, Junhui Liu

**Affiliations:** 1 Department of Cardiology, The First Affiliated Hospital of Xi’an Jiaotong University, Xi’an, Shaanxi 710061, China; 2 Department of Clinical Laboratory, The First Affiliated Hospital of Xi’an Jiaotong University, Xi’an, Shaanxi 710061, China

**Keywords:** Apoptosis, cardiomyocyte damage, CYP11B2, LncRNA GAS5, miR-138-5p

## Abstract

***Objective***: Diabetic cardiomyopathy (DCM) is one of the complications experienced by patients with diabetes. In recent years, long noncoding RNAs (lncRNAs) have been investigated because of their role in the progression of various diseases, including DCM. The purpose of the present study was to explore the role of lncRNA GAS5 in high glucose (HG)-induced cardiomyocyte injury and apoptosis.

***Materials and methods:*** We constructed HG-induced AC16 cardiomyocytes and a streptozotocin (STZ)-induced rat diabetes model. GAS5 was overexpressed and knocked out at the cellular level, and GAS5 was knocked down by lentiviruses at the animal level to observe its effect on myocardial injury. Real-time quantitative polymerase chain reaction (RT-qPCR) was used to detect the expression of GAS5. Cell proliferation and apoptosis after GAS5 knockout were detected by CCK-8, TUNEL, and flow cytometry assays. ELISA was used to detect the changes in myocardial enzyme content in cells and animal myocardial tissues during the action of GAS5 on myocardial injury.

***Results:*** GAS5 expression was up-regulated in HG-treated AC16 cardiomyocytes and the rat diabetic myocardial injury model. The down-regulation of GAS5 could inhibit HG-induced myocardial damage. This work proved that the down-regulation of GAS5 could reverse cardiomyocyte injury and apoptosis by targeting miR-138 to down-regulate CYP11B2.

***Conclusion:*** We confirmed for the first time that the down-regulation of GAS5 could reverse CYP11B2 via the miR-138 axis to reverse HG-induced cardiomyocyte injury. This research might provide a new direction for explaining the developmental mechanism of DCM and potential targets for the treatment of myocardial injury.

## Introduction

Diabetes is a metabolic syndrome that seriously threatens human health worldwide [1]. More than 371 million people are affected currently [2]. The International Diabetes Federation predicts that the number of patients with diabetes will increase to 552 million by 2030 [3]. Diabetes has many complications, and its effect on heart muscle is particularly serious [4,5]. Although its pathogenesis has been proposed from a molecular and cellular perspective and may involve excessive oxidative stress, fibrosis, apoptosis, and inflammation caused by hyperglycemia, controversy remains [6–8]. Currently, specific biomarkers for diabetic cardiomyopathy (DCM) and treatment options that can prevent disease progression do not exist. Therefore, an urgent need to study the pathogenesis of DCM deeply exists. In recent years, noncoding RNAs (ncRNAs) have been found to be widely involved in regulating the disease progression of DCM [9,10].

NcRNAs are widespread in organisms [11]. They are a class of RNA molecules that lack protein-coding function and participate in various physiological and pathological regulatory processes [12]. The number of long ncRNAs (lncRNAs) that play a role in myocardial injury that have been discovered with the development of biochips and sequencing technologies is increasing [13]. A new target for the therapy of DCM can be discovered by exploring the molecular mechanisms, signaling pathways, and biological functions of lncRNAs in diseases; undoubtedly, lncRNAs are becoming an important regulatory element in cardiovascular disease [14]. Apoptosis plays an important role in the occurrence and development of DCM [15]. Studies have reported that lncRNAs are closely related to cardiomyocyte apoptosis [10,16,17]. Yang et al. found that the lncRNA Kcnq1ot1 was involved in many cardiovascular diseases. Kcnq1ot1 expression was increased in hyperglycemia-induced cardiomyocytes and DM mouse heart tissue. When Kcnq1ot1 was silenced, apoptosis and cell death were reduced, thus improving the function and shape of the heart in the body. Therefore, Kcnq1ot1 may be a new therapeutic target for DCM [18]. Competitive endogenous RNA (ceRNA) hypothesis states that lncRNA can not only directly regulate the expression of target genes but also may affect the number of miRNAs by adsorbing the core seed sequences of miRNAs in ncRNAs and subsequently further affecting the level of target gene mRNAs [19]. However, the mechanism underlying the regulatory role of ceRNAs in DCM remains unclear and needs further study.

DCM mainly manifests as myocardial fibrosis, myocardial cell hypertrophy, apoptosis, and metabolic disorders, which ultimately lead to heart failure [20–22]. Aldosterone is the most important and strongest mineralocorticoid in the human body [23]. It can not only cause water and sodium retention, it can also promote collagen synthesis and deposition that lead to cardiac vascular fibrosis and structural remodeling [24]. Its occurrence plays a crucial role in many cardiovascular diseases [25]. Therefore, research on aldosterone and its receptor antagonists has become a hot topic. Aldosterone synthase (CYP11B2) is the catalytic enzyme of the last step in aldosterone synthesis [26]. It belongs to the mitochondrial cytochrome P450 enzyme superfamily [27]. A cytosine and thymine single nucleotide polymorphism is present in the promoter region. Polymorphism plays a role in ventricular remodeling [28]. Therefore, we speculate whether CYP11B2 is involved in the occurrence and development of DCM.

Our study aims to determine the role of lncRNA GAS5 in the progression of DCM and how the mechanism of ceRNA contributes to high glucose (HG)-induced cardiomyocyte injury. We found that the down-regulation of GAS5 can reverse CYP11B2 via the miR-138 axis to reverse HG-induced cardiomyocyte injury. Our findings may provide a new direction for explaining the developmental mechanism of DCM and potential targets for the treatment of myocardial injury.

## Methods

### Animal model

All procedures were approved by the Animal Care and Use Committee of the First Affiliated Hospital of Xi’an Jiaotong University, China, and are in line with the Helsinki Declaration. Twenty-four 7-week-old SPF-grade C57BL/6J mice were obtained from the Military Medical Science Academy Laboratory, Beijing, China, and divided into four groups randomly (Control, Model, Len-si-NC, and Len-si-GAS5), with six mice per group. The mice in the DCM group were injected intraperitoneally with 10 mmol/l streptozotocin (STZ) solution (65 mg/kg) for 5 consecutive days and were given a high-fat, high-sugar diet to establish a type 1 DCM model. An equal amount of citrate buffer was injected intraperitoneally and a common diet was given to the mice in the Control group. One week after STZ injection, the tail vein of each mouse in each group was punctured under fasting conditions to collect blood to measure fasting blood glucose levels. Relevant literature states that a rodent plasma glucose concentration of 16.7 mmol/l indicates that the diabetes model has been successfully modeled. On the basis of the model results, each mouse in the Len-si-NC and Len-si-GAS5 groups was injected with the adenoviruses (1 × 10^11^ PFU, 300 µl/mouse) of Len-si-NC and Len-si-GAS5 through the tail vein, respectively. All animal experiments were conducted in the animal house in the First Affiliated Hospital of Xi’an Jiaotong University. All mice were anesthetized with phenobarbital sodium and then killed with carbon dioxide release devices.

### Cardiomyocyte HG model

AC16 cells were routinely cultured in low-glucose DMEM complete medium (containing 10% fetal bovine serum, 10^5^ U/l penicillin, and 100 mg/l streptomycin). AC16 cells were divided into two groups, namely, the normal glucose control group (5.5 mmol/l glucose) and the HG treatment group (25 mmol/l glucose).

### CCK-8 detection cell activity

AC16 cells in the logarithmic growth phase were collected at a suitable concentration and inoculated into 96-well plates. After different treatments, 10 μl of CCK-8 medium (CK04, Dojindo) was added, and the cells were incubated for 4 h. The absorbance of each well was detected at 490 nm. High absorbance indicated high cell viability. Each group was set up with six replicate wells, and the experiment was repeated three times independently.

### Cell transfection

SiRNA was designed and synthesized by Beijing Qingke Biotechnology Co., Ltd. Cardiomyocytes were transfected with siRNA as follows: primary cardiomyocytes were spread into a six-well plate. When the cell fusion reached 30%, Lipofectamine™ 2000 (11668027, Invitrogen) was used to transfer siRNA into cells in reference to the instructions provided with the Lipofectamine™ 2000 transfection reagent. Cells transfected with siRNA were divided into the si-GAS5 group, FGF13-si2 group, FGF13-si3 group, and NC-siRNA negative control group. The medium was changed 6 h after transfection.

### Real-time quantitative polymerase chain reaction

TRIzol (15596018, Invitrogen) method was used to extract total RNA for reverse transcription. A 20-μl reaction system was used for real-time quantitative polymerase chain reaction (RT-qPCR). The reaction system comprised 10 μl of SYBR Premix Ex Taq™ II (2×, RR420L, Takara), 0.4 μl of ROX Reference Dye (50×, 31110, Lumiprobe), 2 μl of reverse transcription product (18090010, Invitrogen), 6 μl of ddH_2_O, and 0.8 μl each of 1 ng/ml upstream and downstream primers. The reaction conditions were as follows: predenaturation at 95°C for 30 s, denaturation at 95°C for 5 s, annealing at 55°C for 30 s, and extension at 72°C for 30 s for a total of 40 cycles. The relative amount of gene expression was calculated in accordance with the comparison method, and the relative fold change of gene expression was calculated by using the formula 2^–ΔΔC_t_^.

### Flow cytometry

The cells in each group were incubated with trypsin, centrifuged for 48 h, washed with prechilled phosphate-buffered saline, and resuspended. In accordance with the instructions of the Annexin V-FITC/PI Apoptosis Detection Kit, 5 μl of Annexin V-FITC and 10 μl of PI were added to the cells. The cells were mixed gently and incubated at room temperature in the dark for 15 min. Flow cytometry was used to detect apoptosis. The experiment was repeated three times with three complex holes set each time.

### Lactate dehydrogenase

After culturing the cells in each group for 48 h, the culture supernatant was collected, processed in accordance with the instructions of the lactate dehydrogenase (LDH) kit, and finally mixed. After standing at room temperature for 5 min, the absorbance was measured at 440 nm. The experiment was repeated three times. Each set involved three complex holes.

### TUNEL detection

Cells were seeded in six-well plates and stimulated in groups. The specific experimental steps were carried out in accordance with the instruction manual of the kit (A23210, Invitrogen). The cells with green nuclei represent positive. At least 300 cells were counted in each well to calculate the percentage of positive cells.

The anterior wall tissue of the left ventricle of each group of mice was taken for routine paraffin embedding and sectioning. After dewaxing and rehydration, apoptosis was detected via the TUNEL method (C10617, Invitrogen). Treatment was carried out in strict accordance with the kit instructions. Images were collected with a laser confocal microscope. Ten high-magnification fields were randomly selected for each slice, and the apoptosis rate was calculated as follows: apoptotic nuclei/total nuclei × 100%. Green indicated apoptotic nuclei, and blue indicated normal nuclei.

### ELISA detection of low-density lipoprotein, TNF-ɑ, IL-1β, IL-6, SOD, and MDA levels in cell supernatants and mouse serum

AC16 cells in the logarithmic growth phase were added to 96-well plates (10000 cells/well). After 48 h, the cell supernatant was collected. Changes in cytokine low-density lipoprotein (LDL) (EK-3307, Azeroth), TNF-ɑ (BMS607-3TWO, Invitrogen), IL-1β (EMC001b.96, NeoBioscience), IL-6 (BMS603-2, Invitrogen), SOD (DL-SOD-Ra, Dldevelop), and MDA (YM-4169B, Yuanmu) levels were detected by following the instructions included with the kits.

After anesthetization, mice were bled via ventricular puncture. The blood sample was placed at 4°C for 12 h and centrifuged at 4°C and 3000 rpm for 15 min with the centrifugal radius of 12 cm. The supernatant was then collected. Changes in cytokine LDL, TNF-ɑ, IL-1β, IL-6, SOD, and MDA levels were determined in accordance with the kits’ instructions.

### Statistical analysis

Data were expressed as mean ± SEM and were obtained from five to ten separate experiments. Statistical significance was determined by using Student’s *t* test or one-way ANOVA followed by Tukey’s test. *P*<0.05 was considered statistically significant.

## Results

### HG-induced cardiomyocyte injury

**Figure 1:**
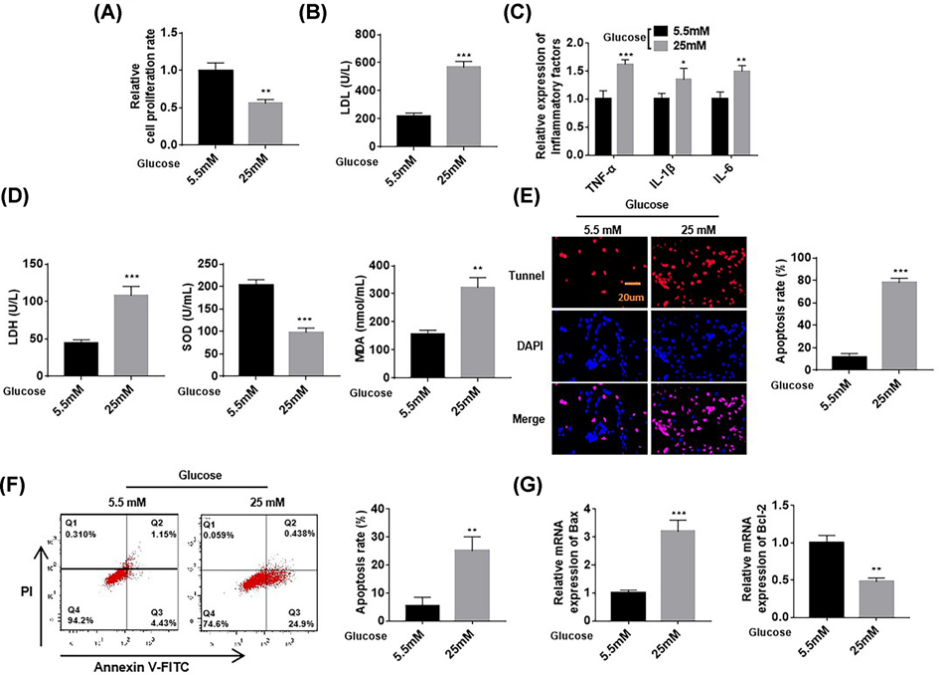
HG-induced cardiomyocyte injury Compared with the normal glucose treatment group (5.5 mM), AC16 cells in the 25 mM HG treatment showed decreased cell survival rates (**A**) and increased LDL (**B**), TNF-α, IL-1β, and IL-6 (**C**). (**D**) Compared with cells under 5.5 mM treatment, AC16 cells treated with 25 mM HG showed increased LDH, decreased SOD, and increased MAD. (**E**–**G**) TUNEL, flow cytometry, and RT-qPCR analyses were performed to detect cardiomyocyte apoptosis. Cell apoptosis in the 25 mM HG treatment group increased compared with that in the 5.5 mM group. *n*=6, *P<0.05, **P<0.01, ***P<0.001.

First, we used the CCK-8 method to evaluate the survival of AC16 cells after HG treatment. The results are shown in Figure 1A. The cell survival rate of AC16 cells after 48 h of 25 mM HG treatments decreased (0.56 ± 0.05, *P*<0.01) compared with that of the cells under normal glucose treatment (5.5 mM). We performed ELISA to detect the levels of LDL; inflammation-related factors TNF-ɑ, IL-1β, and IL-6; LDH; and oxidative stress indicators SOD and MAD in AC16 cardiomyocytes under 25 mM HG treatment. The results showed that compared with cells under normal glucose treatment (5.5 mM), AC16 cells after 48 h of 25 mM HG treatment showed increased levels of intracellular LDL (567 ± 42, *P*<0.01), inflammation-related factors (TNF-α (1.62 ± 0.08, *P*<0.01), IL-1β (1.35 ± 0.20, *P*<0.01), and IL-6 (1.50 ± 0.10, *P*<0.01). LDH content increased, SOD content decreased, and MDA content increased. Myocardial cells exhibited increased inflammatory response and oxidative stress levels (Figure 1B–D) after HG treatment. TUNEL results showed that apoptosis in the 25 mM HG treatment group had increased (12 ± 3) compared with that in the 5.5 mM sugar treatment group (78 ± 4) (Figure 1E, *P*<0.01). Flow cytometry results also showed that apoptosis in the 25 mM HG treatment group had increased compared with that in the 5.5 mM sugar treatment group (Figure 1F). The results of RT-qPCR showed that Bax mRNA expression increased and Bcl-2 mRNA expression decreased (Figure 1G). The above results indicated that HG promoted apoptosis.

### HG induced the high expression of lncRNA GAS5

**Figure 2:**
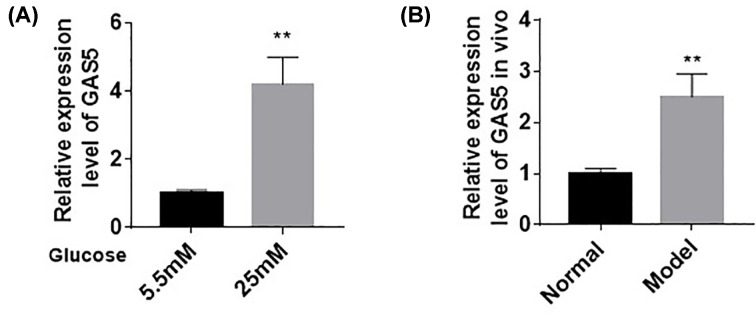
HG induced the high expression of lncRNA GAS5 (**A**,**B**) *In vivo* and *in vitro* RT-qPCR results showed that GAS5 expression in cardiomyocytes increased after HG treatment. *n*=6, **P<0.01.

After successfully inducing HG damage in myocardial cells, we detected the expression of GAS5 in AC16 cardiomyocytes after HG treatment by using RT-qPCR. *In vivo* (4.2 ± 0.8) and *in vitro* (2.5 ± 0.5) results showed that GAS5 expression in cardiomyocytes increased after HG treatment (Figure 2A,B, *P*<0.01). This result indicated that HG stimulation could induce GAS5 expression in cardiomyocytes.

### Knockdown of GAS5 at the cellular level inhibited HG-induced myocardial damage

**Figure 3:**
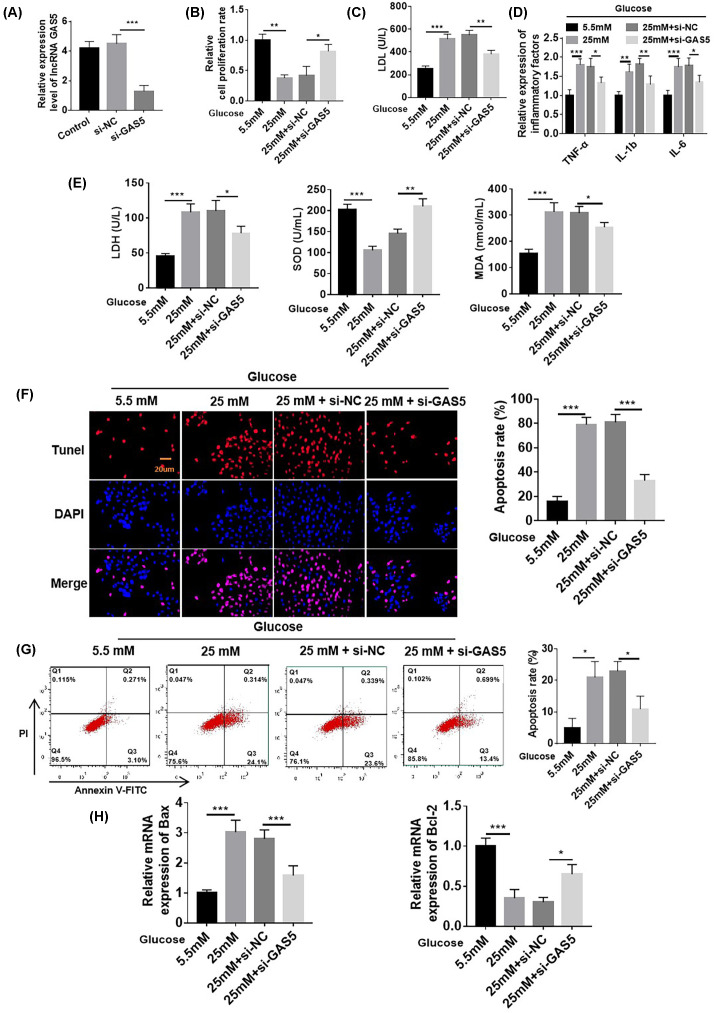
Knockdown of GAS5 at the cellular level inhibited myocardial damage induced by HG (**A**–**D**) Compared with cells in the 5.5 mM treatment group, AC16 cells under 25 mM HG treatment showed decreased survival rates and increased LDL, TNF-α, IL-1β, and IL-6 levels; these effects were abolished by si-GAS5. (**E**) Compared with cells in the 5.5 mM treatment group, AC16 cells under 25 mM HG treatment showed increased LDH, decreased SOD, and increased MAD; these effects were abolished by si-GAS5. (**F–H**) TUNEL, flow cytometry, and RT-qPCR analyses were used to detect cardiomyocyte apoptosis. Cell apoptosis in the 25 mM HG treatment group increased relative to that in the 5.5 mM group; this effect was abolished by si-GAS5. *n*=6, *P<0.05, **P<0.01, ***P<0.001.

We used RT-qPCR to detect GAS5 expression in cardiomyocytes after the transfection of specific small-RNA interference sequences (si-GAS5). The results are shown in Figure 3A. After transfection with si-GAS5, the expression of GAS5 in AC16 cardiomyocytes decreased (1.3 ± 0.38), indicating that small-RNA interference technology could effectively down-regulate the expression of GAS5 in AC16 cardiomyocytes. We found that the successful transfection of si-GAS5 down-regulated the expression of GA16 in AC16 cardiomyocytes in the sugar-treated group and that the survival rate of cardiomyocytes increased; the content of LDL decreased; the expression of inflammation-related factors (TNF-α, IL-1β, and IL-6) and the content of LDH and MAD related to oxidative stress decreased; and the content of SOD increased (Figure 3B–E). Similarly, TUNEL and flow cytometry showed that compared with that in the HG treatment group, cardiomyocyte apoptosis decreased in the si-GAS5 treatment group. Bax mRNA expression decreased, and Bcl-2 mRNA expression increased (Figure 3F–H).

### GAS5 knockdown could inhibit myocardial injury in the animal model

**Figure 4:**
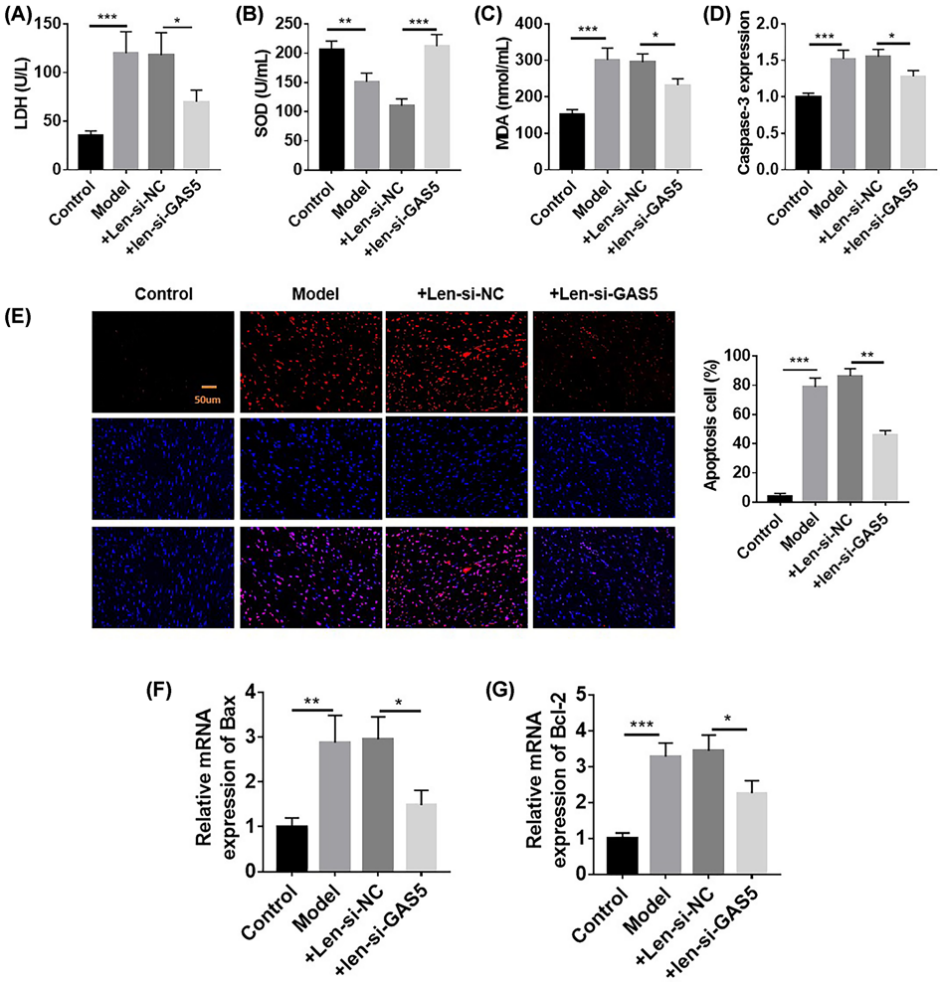
Knockdown of GAS5 could inhibit myocardial injury in the animal model (**A**–**C**) Compared with the control, the diabetic animal model showed increased LDH, decreased SOD, and increased MAD; these effects were abolished by si-GAS5. (**D**–**G**) Cell apoptosis increased in the diabetic animal model compared with that in the control; this effect was abolished by si-GAS5. *n*=6, *P<0.05, **P<0.01, ***P<0.001.

We constructed heart-specific GAS5 knockdown mice via lentiviral technology. The results obtained with this model were similar to those obtained with the cell experiment. Compared with those in the model group, serum LDH, MAD, and Caspase-3 levels decreased and SOD levels increased in the GAS5 knockdown group, indicating that oxidative stress in the rat heart was reduced after GAS5 knockdown (Figure 4A–D). TUNEL test results showed that compared with that in the model group, the apoptosis of cardiomyocytes in the GAS5 knockdown group decreased. TUNEL test results showed that compared with that in the HG treatment group, cardiomyocyte apoptosis decreased in the si-GAS5 treatment group. Flow cytometry results also showed that compared with that in the HG treatment group, cardiomyocyte apoptosis decreased in the si-GAS5 treatment group (Figure 4E). RT-qPCR detection results showed that compared with that in the HG treatment group, Bax mRNA expression decreased and Bcl-2 mRNA expression increased in the si-GAS5 treatment group (Figure 4F–G). The above results indicated that GAS5 knockdown could antagonize HG-induced apoptosis.

### GAS5 induced cardiomyocyte damage by acting as the ceRNA of miR-138

**Figure 5:**
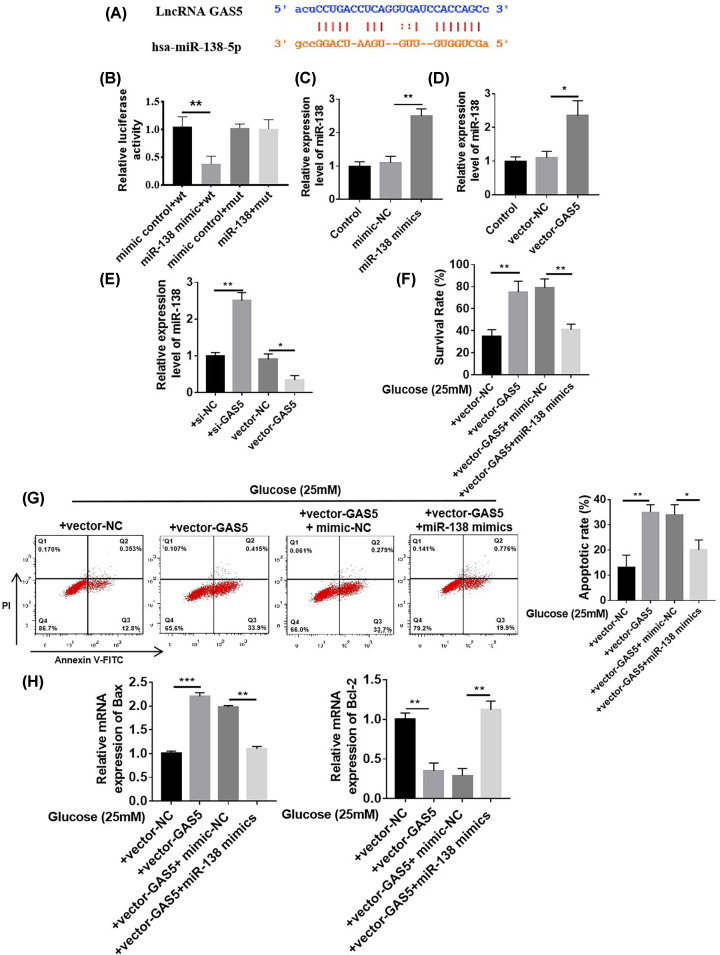
GAS5 induced cardiomyocyte damage by acting as a ceRNA of miR-138 (**A**) Predicted GAS5 and miR-138 binding sites. (**B**) Luciferase value decreased after the cotransformation of GAS5 with the wild-type miR-138 mimic but did not significantly decrease after cotransformation with the mutant miR-138 mimic, **P<0.01 vs. mimic control + wt. (**C**) miR-138 expression increased after cardiomyocytes were transfected with the miR-138 mimic, **P<0.01 vs. mimic-NC. (**D**) GAS5 expression increased after cardiomyocytes were transfected with vector-GAS5, **P<0.01 vs. vector-NC. (**E**) RT-qPCR results showed that the expression of miR-138 in cardiomyocytes in the si-GAS5 group increased, whereas that in the cardiomyocytes in the vector-GAS5 group decreased, **P<0.01 vs. si-NC, *P<0.05 vs. Vector-NC. (**F**–**H**) Under HG treatment, compared with the vector, vector-GAS5 promoted apoptosis, whereas the miR-138 mimic could reverse GAS5 induced-damage. *n*=6, ***P<0.001 vs. Vector-NC, *P<0.05 vs. Vector-NC + mimic-NC.

GAS5 and miR-138 binding sites were predicted (Figure 5A) by using the bioinformatics tool starBase v2.0 (http://starbase.sysu.edu.cn/index.php) and LncBase Predicted v.2 tools (http://carolina.imis.athena-innovation.gr/diana_tools/ web/index.php? r = lncbas ev2/index-predicted). The results of luciferase experiments showed that the luciferase value decreased after the cotransformation of GAS5 with wild-type miR-138 mimic but did not significantly decrease after cotransformation with the mutant miR-138 mimic (Figure 5B). These results confirmed the possible specific binding of GAS5 to miR-138. After transfecting cardiomyocytes with the miR-138 mimic, the expression of miR-138 increased (Figure 5C). GAS5 levels increased after cardiomyocytes were transfected with vector-GAS5 (Figure 5D). We observed the changes in miR-138 expression after transfecting cardiomyocytes with si-GAS5 and vector-GAS5 to further verify the existence of mutual inhibition between GAS5 and miR-138. RT-qPCR results showed that the expression of miR-138 in the cardiomyocytes of the si-GAS5 group increased, whereas the expression of miR-138 in the cardiomyocytes of the vector-GAS5 group decreased (Figure 5E). TUNEL results showed that vector-GAS5 promoted apoptosis in the HG treatment group relative to that in the vector group, whereas the miR-138 mimic could reverse GAS5-induced apoptosis (Figure 5F). Flow cytometry results also showed that under HG treatment, apoptosis was promoted in the vector-GAS5 group relative to that in the vector group, whereas GAS5-induced apoptosis was reversed in the miR-138 mimic group (Figure 5G). RT-qPCR results showed that compared with the vector group, the vector-GAS5 group exhibited the increased expression of apoptosis-related protein Bax mRNA and the decreased expression of Bcl-2 mRNA, and the miR-138 mimic could reverse the damage of GAS5 (Figure 5H). The above results indicated that GAS5 acted as a miR-138 ceRNA to promote cardiomyocyte damage.

### GAS5/miR-138 axis regulated cardiomyocyte injury though CYP11B2

**Figure 6:**
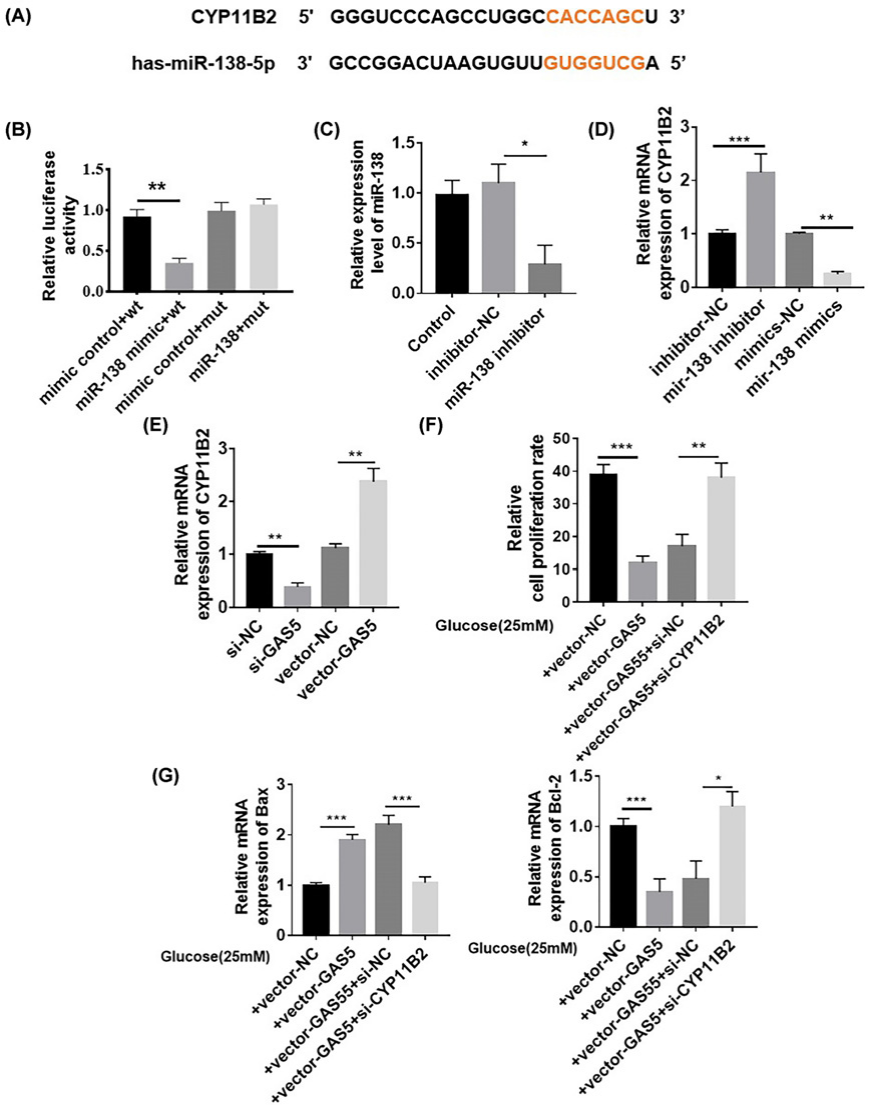
GAS5/miR-138 axis regulated cardiomyocyte injury though CYP11B2 (**A**) Predicted CYP11B and miR-138 binding sites. (**B**) Luciferase experimental results showed that the luciferase value decreased after the cotransformation of CYP11B2 with the wild-type miR-138 mimic but did not significantly decrease after cotransformation with the mutant miR-138 mimic, **P<0.05 vs. mimic control + wt. (**C**) miR-138 expression decreased after cardiomyocytes were transfected with miR-138 inhibitor, *P<0.05 vs. Inhibitor-NC. (**D**) After the expression of miR-138 was inhibited with an inhibitor, the expression of CYP11B2 mRNA increased; after miR-138 mimics were transfected into AC16 cells, the expression of CYP11B2 mRNA decreased, ***P<0.001 vs. inhibitor-NC, **P<0.01 vs. miR-138 mimics. (**E**) CYP11B2 mRNA expression in AC16 cardiomyocytes decreased after si-GAS5 transfection, whereas CYP11B2 mRNA expression in AC16 cardiomyocytes increased after transfection with vector-GAS5, **P<0.01 vs. si-NC or vector-NC. (**F**–**G**) In AC16 cardiomyocytes under HG treatment, GAS5 overexpression reduced cell activity, whereas si-CYP11B2 could antagonize the effect of GAS5, *P<0.05, *n*=6.

TargetScan database predictive analysis revealed the presence of an miR-138 binding site in the 3′UTR region of CYP11B2 (Figure 6A). Luciferase experimental results showed that luciferase activity decreased after the cotransformation of CYP11B2 with the wild-type miR-138 mimic but did not significantly decrease after cotransformation with the mutant miR-138 mimic (Figure 6B). After inhibiting the expression of miR-138 with inhibitors, the expression of CYP11B2 mRNA increased; after miR-138 mimics were introduced into AC16 cells, the expression of CYP11B2 mRNA decreased, indicating that miR-138 could inhibit the expression of CYP11B2 (Figure 6C,D). Similarly, the expression of CYP11B2 mRNA in AC16 cardiomyocytes decreased after transfection with si-GAS5, whereas the expression of CYP11B2 mRNA in AC16 cardiomyocytes increased after transfection with vector-GAS5 (Figure 6E). In AC16 cardiomyocytes treated with HG, GAS5 overexpression could reduce cell activity, whereas si-CYP11B2 transfection could antagonize the effect of GAS5 (Figure 6F). Furthermore, RT-qPCR results indicated that compared with those under + vector-GAS5 + si-NC treatment, the expression of apoptosis-related protein Bax mRNA decreased and the expression of Bcl-2 mRNA increased under si-CYP11B2 treatment (Figure 6G).

## Discussion

The present study showed that GAS5 expression was up-regulated in HG-treated AC16 cardiomyocytes and a rat diabetic myocardial injury model. The down-regulation of GAS5 could inhibit HG-induced myocardial injury. These results clarified that the down-regulation of GAS5 could reverse the molecular mechanism of myocardial cell injury and apoptosis by targeting miR-138 to down-regulate CYP11B2 further.

LncRNAs can regulate the expression of genes at the transcriptional and post-transcriptional levels and play an important role in physiological processes [29]. Some functions of lncRNAs include structural scaffolding, RNA processing, apoptosis and cell invasion regulation, and chromatin rearrangement [30]. The lncRNA SENCR is highly expressed in endothelial cells, vascular smooth muscle cells, and aortic tissues in the heart [31,32]. Silencing SENCR down-regulates the expression of some vascular smooth muscle contractile proteins. The lncRNA Mhrt is rich in ncRNA and is unique to human heart tissue. Under normal circumstances, Mhrt can hinder cardiac hypertrophy and heart failure [33]. Dong et al. found that in liver fibrosis, the overexpression of the lncRNA GAS5 can inhibit the activation of primary hepatic stellate cells *in vitro* and reduce the accumulation of collagen in fibrotic liver tissues [34]. In addition, the existing literature has proven that GAS5 is expressed at low levels in many malignant tumors, exhibits tumor-suppressing characteristics, and participates in cell cycle regulation [35]. The results of *in vitro* and *in vivo* animal experiments in this study showed that the knockdown of lncRNA GAS5 can inhibit HG-induced cardiomyocyte inflammation, apoptosis, and oxidative stress injury, indicating that HG may induce cardiomyocyte injury through the lncRNA GAS5 signaling factor.

LncRNAs and microRNAs play a key role in regulating cell growth and apoptosis [36,37]. Recent studies have shown that lncRNAs regulate gene expression and can interact with microRNAs [38]. Leonaedo et al. reported that lncRNAs can regulate the expression level of miR-489 in the myocardial tissue of patients with myocardial hypertrophy, thereby reducing myocardial hypertrophy [39]. In a study on patients with breast cancer, Zhang et al. found that the level of miR-21 in cancerous tissue was higher than that in adjacent tissues and could reduce the level of GAS5, and interference with GAS5 could increase the expression of miR-21 [40]. Similarly, this study found that after knocking down GAS5 in myocardial cells, miR-138 expression increased, and the overexpression of the miR-138 mimic could reverse GAS5-induced myocardial injury.

The renin–angiotensin–aldosterone system (RAAS) plays an important role in the progression of diabetes to heart failure and DCM. Studies have demonstrated that the activation of the RAAS system is closely associated with cardiac hypertrophy, fibrosis, and myocardial injury in patients with diabetes. The stimulation of angiotensin receptor 1 by angiotensin II exerts direct effects on the myocardial cells and fibroblasts of the heart. It increases collagen synthesis; reduces collagen breakdown; causes cardiac hypertrophy and fibrosis; and leads to decreased ventricular compliance and systolic and diastolic dysfunction. Aldosterone can promote the mRNA expression of the collagen gene in cardiomyocytes and also cause myocardial remodeling through other ways, such as promoting inflammation and oxidative stress and damaging vascular endothelial cell function [41]. Therefore, studying the influencing factors of aldosterone synthesis is beneficial for the targeted blocking of aldosterone synthesis and reducing aldosterone levels and is conducive to reducing heart failure remodeling. CYP11B2 is the main restriction enzyme in aldosterone synthesis [42]. Studies have shown that the protein kinase A signaling pathway can induce the increased expression of genes related to steroid hormone synthesis, including CYP11B2 [43]. Steroidogenic factor-1 (SF-1), an important transcriptional regulator, can act on the 344 position of the CYP11B2 gene. After T/C mutation, SF-1 can easily change the expression activity of CYP11B2 in TT and TC genotypes relative to that in CC genotypes; it can also change the sensitivity of CYP11B2 to AT-II [44]. This study found that the expression of miR-138 in AC16 cardiomyocytes further increased after transfection with si-GAS5; this effect subsequently caused the expression of CYP11B2 mRNA to decrease, thereby reversing HG-induced cardiomyocyte damage. This result suggested putative advantages to blocking GAS5 over direct aldosterone blockade to prevent the development of DCM.

The present study confirmed for the first time that the down-regulation of GAS5 could reverse CYP11B2 via the miR-138 axis to reverse HG-induced myocardial cell injury. Our findings might provide a new direction for explaining the developmental mechanism of DCM and potential targets for the treatment of myocardial injury.

## Data Availability

All data are available upon request.

## Abbreviations

ceRNA: competitive endogenous RNA
DCM: diabetic cardiomyopathy
HG: high glucose
LDH: lactate dehydrogenase
LDL: low-density lipoprotein
lncRNA: long noncoding RNA
ncRNA: noncoding RNA
RAAS: renin–angiotensin–aldosterone system
RT-qPCR: real-time quantitative polymerase chain reaction
SF-1: steroidogenic factor-1
STZ: streptozotocin
